# Recent Advances in Molten Salt-Based Nanofluids as Thermal Energy Storage in Concentrated Solar Power: A Comprehensive Review

**DOI:** 10.3390/ma17040955

**Published:** 2024-02-19

**Authors:** Fahim Mahtab Abir, Qutaiba Altwarah, Md Tasnim Rana, Donghyun Shin

**Affiliations:** 1 School of Engineering and Technology, Central Michigan University, Mt. Pleasant, MI 48859, USA; abir1f@cmich.edu (F.M.A.); altwa1qm@cmich.edu (Q.A.); 2 Bangladesh Council of Scientific and Industrial Research, Dhaka 1205, Bangladesh

**Keywords:** molten salt nanofluid, thermal energy storage, concentrated solar power, specific heat capacity, thermal conductivity, material characterization, thermal analysis

## Abstract

This study critically reviews the key aspects of nanoparticles and their impact on molten salts (MSs) for thermal energy storage (TES) in concentrated solar power (CSP). It then conducts a comprehensive analysis of MS nanofluids, focusing on identifying the best combinations of salts and nanoparticles to increase the specific heat capacity (SHC) efficiently. Various methods and approaches for the synthesis of these nanofluids are explained. The article presents different experimental techniques used to characterize nanofluids, including measuring the SHC and thermal conductivity and analyzing particle dispersion. It also discusses the challenges associated with characterizing these nanofluids. The study aims to investigate the underlying mechanisms behind the observed increase in SHC in MS nanofluids. Finally, it summarizes potential areas for future research, highlighting crucial domains for further investigation and advancement.

## 1. Introduction

### 1.1. Concentrated Solar Power (CSP) with Thermal Energy Storage (TES)

**Figure 1:**
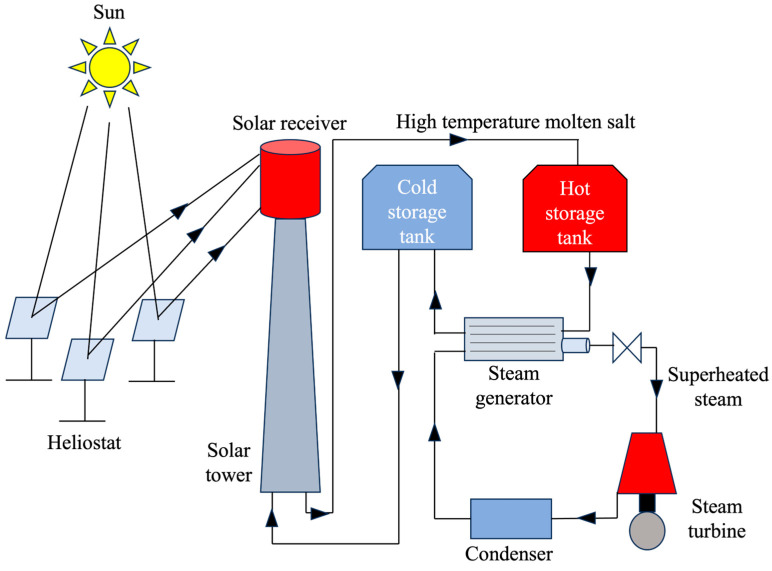
Schematic of a concentrated solar power plant.

Concentrated solar power (CSP) technology offers a financially viable way to harness renewable energy for electricity generation. It utilizes an array of solar receivers (lenses, mirrors, or heliostats) to collect and focus incoming solar energy [1]. This concentrated energy is then directed towards a solar tower or collection unit. A heat transfer fluid (HTF) system carries the collected thermal energy, with the option of storing excess capacity in a thermal energy storage (TES) device. Finally, a power conversion module, typically employing turbines (Rankine cycle) or engines (Stirling cycle), converts the stored thermal energy into usable electricity. Figure 1 illustrates the schematic of a typical CSP plant.

The efficiency of the Rankine cycle in terms of thermodynamics is mostly dependent on the temperature differential that exists between the entrance of the turbine (which is hot) and the condenser (which is cool). Because it is highly costly to lower the temperature of the condenser, raising the temperature at the point of entry is the critical factor in improving the overall system efficiency of the CSP. The material characteristics of the TES set a limit on this temperature.

### 1.2. Importance of Nanofluids

Nanofluids are created by dispersing nanoparticles or nanosheets in base fluids, such as water, ethylene glycol (EG), a transformer oil, paraffin oil, vegetable oil, or a water–EG mixture [2]. Two common preparation methods are physical mixing and chemical reduction [3]. Incorporating nanoparticles into a base fluid as a colloidal suspension significantly improves the thermophysical properties of the base fluid. These advancements have found application across various industries, including HVAC, thermal power generation, transportation, microelectronics, aerospace, and manufacturing [4]. In thermal devices, nanofluids are replacing traditional heat transport media with low thermal conductivity to achieve higher thermal efficiency. By adding nanoparticles to conventional fluids, the thermal conductivity is enhanced, leading to improved efficiency [5]. For instance, Eastman et al. [6] found that a 0.3 vol.% concentration of Cu nanoparticle/EG nanomaterial increased the effective thermal conductivity by 40%. Similar observations of increased thermal conductivity are reported elsewhere [7,8,9,10]. However, some studies [11,12] have shown that nanoparticle dispersions in common base liquids, especially water-based ones, can reduce the specific heat capacity (SHC). Conversely, recent studies [13,14,15,16,17,18,19] have documented an increase in SHC for oxide nanoparticle dispersions in ionic liquids (molten salts).

### 1.3. Importance of Molten Salt (MS) Nanofluid for CSP

Molten salt (MS) mixtures are gaining popularity as heat transfer base fluids for their ability to function well across a wider temperature range, boosting the process efficiency. Carnot’s efficiency principle dictates that higher operating temperatures lead to higher efficiency in power production systems [20]. Conventional heat transfer fluids, such as water, oil, or ethylene glycol, often have a low boiling point (less than 400 °C), which renders them incompatible with CSP systems that operate at higher temperatures (above 400 °C). To overcome this limitation, MSs, which are made up of nitrate, carbonate, and chloride salts, might be used to store and transport thermal energy. Several benefits arise from the use of MSs as TES materials [21]: (1) higher temperature stability in molten salts allows for an increase in the operating temperature of CSP systems, resulting in a more efficient thermodynamic cycle; (2) the lower cost of MSs compared to traditional TES materials could substantially reduce the CSP system’s expenses; (3) the MSs present minimal environmental risk, potentially avoiding significant restoration costs. However, the use of MSs in TES is hindered by their inherently low thermal conductivity and SHC [21].

MSs, particularly nitrate-based, carbonate-based, or chloride-based mixtures, have emerged as promising TES for high-temperature CSP plants [22]. Researchers have explored the enhancement of the thermal properties of MSs by incorporating nanoparticles. Studies have shown that MS-based nanofluids exhibit improved heat transfer compared to pure MSs, primarily due to increased thermal conductivity and SHC [23,24]. However, the effectiveness of these nanofluids relies on the careful selection of the nanoparticles, their concentrations, and their ability to maintain stable dispersion within the MS matrix [25].

### 1.4. Types of MS Nanofluid Used in Previous Studies

This innovative and cost-effective solution involves enhancing the thermophysical properties of molten salts by injecting them with minute quantities of nanoparticles, creating a stable, homogeneous colloidal suspension. These nanofluids, defined as engineered colloidal suspensions of nanoparticles (1–100 nm) in a base fluid, are currently being investigated by researchers as a means to boost the overall efficiency of CSP systems.

Table 1 shows SHC enhancements for various combinations of base salt systems and nanoparticles documented in previous studies. CSP systems can utilize single, binary, ternary, or even quaternary salts as the base fluid. Among these, NaNO_3_-KNO_3_ (60:40 weight ratio), also known as solar salt, is the most popular choice. The introduction of nanoparticles has emerged as a promising approach to enhance the SHC of molten salts, thereby improving their heat storage and transfer capabilities. Extensive research has been conducted on various categories of nanoparticles, including carbon nanotubes, graphene, metal oxides, and metallic particles, to achieve this goal. These nanoparticles possess unique characteristics, such as high surface-to-volume ratios and efficient heat conduction, enabling them to readily absorb and release heat. Integrating nanoparticles into molten salts holds significant potential in boosting the thermal energy storage capacity and overall efficiency of CSP systems, paving the way for the advancement of sustainable energy technologies.

This review focuses on the fundamental characteristics of nanoparticles and their effect on molten salts (MSs) for thermal energy storage (TES) in concentrated solar power (CSP). It then performs a thorough investigation of MS nanofluids, with an emphasis on determining the most effective salt and nanoparticle combinations to effectively increase the specific heat capacity (SHC). Various techniques and approaches to manufacturing these nanofluids are discussed. The article describes many experimental methodologies for the characterization of nanofluids, such as measuring the SHC and thermal conductivity and assessing the particle dispersion. It also examines the difficulties connected with characterizing these nanofluids. The study’s goal is to understand the underlying processes behind the observed rise in SHC in MS nanofluids. In conclusion, it provides a synopsis of prospective topics for future study, directing attention to essential domains that need more exploration and development.

## 2. Synthesis Methods and Techniques

**Figure 2:**
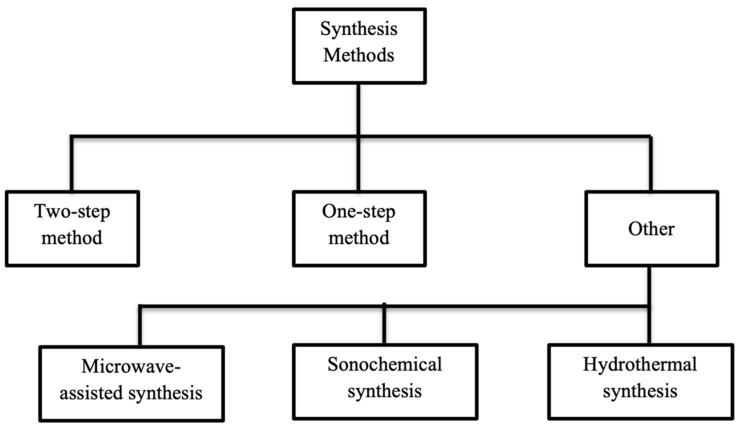
Synthesis methods for nanofluid preparation.

Figure 2 depicts the schematic showing the different synthesis methods associated with nanofluid preparation.

### 2.1. Two-Step Method

**Figure 3:**
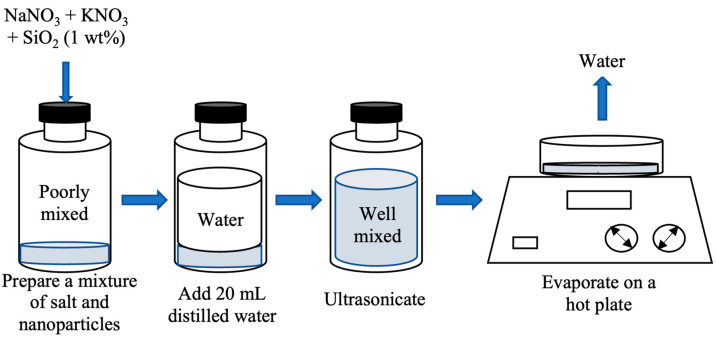
LS (two-step) method for nanofluid synthesis. Reprinted form Ref. [31].

The most common method of preparing molten salt nanofluids is the liquid solution (LS) method, also known as the two-step method. This flexible and scalable approach allows for the synthesis of a wide range of nanofluids with diverse properties. The two-step process begins with preparing the nanoparticles, which can involve chemical synthesis, physical synthesis, or techniques using ionic liquids. The nanoparticles are then dispersed evenly throughout the molten salt using ultrasonic agitation, mechanical stirring, or high-shear mixing. Finally, a surfactant or coating is added to prevent the nanoparticles from clumping and precipitating out of the molten salt. This two-step method offers several advantages. It is straightforward and easy to understand, allowing for clear control over the nanofluid properties. Its flexibility enables the creation of diverse nanofluids with tailored characteristics. Additionally, it is scalable, meaning that large quantities of nanofluids can be produced for further research or applications. As an example, Shin and Banerjee [30] employed the two-step method to prepare their MS nanofluids for the first time. Their process involved measuring the required amounts of MS powder and nanoparticles, mixing them with distilled water, and subjecting the mixture to ultrasonic treatment for 2 h. The resulting water solution was then rapidly evaporated using a hot plate, leaving behind the ready-to-use MS nanofluid for further testing. This specific example is illustrated in Figure 3.

### 2.2. One-Step Method

Another method of preparing molten salt nanofluids is the in-situ, or one-step, approach. This simpler and cheaper alternative to the two-step method dissolves a precursor compound into nanoparticles by heating a salt solution to high temperatures. The resulting nanoparticles are then dispersed throughout the salt solution. While the one-step method offers advantages in terms of time and cost, it can be more challenging to control and less reproducible compared to the two-step approach. The following is a simplified overview of the one-step process: (1) combine molten salt and precursor compound; (2) heat the mixture to a high temperature, causing the precursor to decompose and form nanoparticles; (3) disperse the nanoparticles throughout the molten salt.

**Figure 4:**
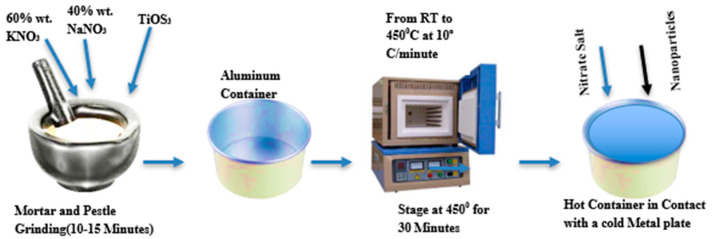
In-situ (one-step) method for nanofluid synthesis.

The in-situ fabrication of nanoparticles within the MS is schematically illustrated in Figure 4. This specific example incorporates 3.0 wt% of TiOSO_4_ as a precursor into an MS nanofluid composed of the eutectic mixture NaNO_3_-KNO_3_ at a 60:40 weight ratio [32]. While existing methods might not be entirely cost-effective for large-scale production, the one-step approach is known for its economic advantage [33,34]. This method often employs techniques like microwave-assisted synthesis (heating molten salt and precursor with microwaves), hydrothermal synthesis (using high pressure and temperature in a sealed container), or sonochemical synthesis (sonication of the compound with ultrasound). 

## 3. Experimental Techniques for Evaluation of Thermophysical Properties and Characterization of MS-Based Nanofluids

### 3.1. Thermal Characterization

#### 3.1.1. DSC

**Figure 5:**
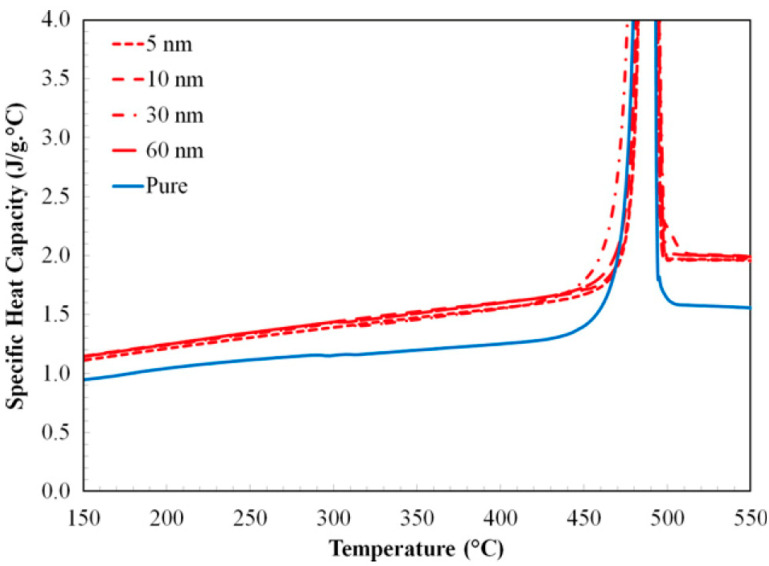
Specific heat capacity changes across a wide temperature range for both pure eutectic and nanomaterials (with sizes of 5 nm, 10 nm, 30 nm, and 60 nm). Reprinted with permission from Ref. [37] Copyright 2013 Elsevier.

The accurate characterization of thermophysical properties and stability is crucial for MS-based nanofluids, and various experimental techniques are employed for this purpose [35]. Among the key properties, the SHC plays a vital role in determining the nanofluids’ ability to store and transfer thermal energy. Differential scanning calorimetry (DSC) has emerged as a popular technique for the measurement of the SHC [36]. It involves subjecting the nanofluid to controlled heating and analyzing the heat flow rate, allowing the subsequent calculation of the SHC from these measurements. Figure 5, adapted from Tiznobaik and Shin [37], illustrates the temperature dependence of the SHC for pure binary carbonate eutectic and various binary carbonate nanofluids containing silica nanoparticles of different sizes (5 nm, 10 nm, 30 nm, and 60 nm) over a range of 150 °C to 550 °C. As shown, the nanofluid with 5 nm nanoparticles (99% salt eutectic + 1% 5 nm silica) exhibited an average SHC enhancement of 24% compared to the pure eutectic one. Similar trends were observed for larger nanoparticles, with average increases of 26% for 10 nm, 23% for 30 nm, and 26% for 60 nm. 

DSC offers numerous advantages in characterizing materials, particularly in small quantities (<10 mg). It accurately measures the phase transitions, heat capacity, and temperature, making it a cost-effective way to determine key thermal properties. Additionally, DSC can detect subtle phase transitions that other techniques might miss. However, DSC also comes with some limitations. The process is destructive, meaning that the sample cannot be reused. Moreover, discrepancies in DSC results can occur between different research facilities due to variations in equipment and calibration. Additionally, DSC provides limited structural information about the material. Despite these limitations, DSC has a wide range of applications. It detects phase transitions in polymeric materials, such as melting, crystallization, and glass transition. It quantifies the heat capacity of both individual substances and mixtures. It determines the heat of fusion and solidification to understand enthalpy changes. 

#### 3.1.2. Thermogravimetric Analysis (TGA)

**Figure 6:**
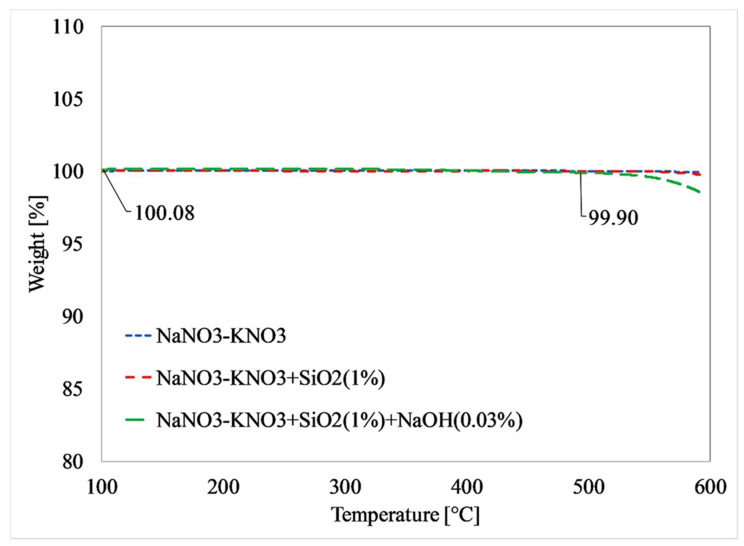
A thermogravimetric analysis shows no moisture presence or chemical reaction in NaNO_3_-KNO_3_, NaNO_3_-KNO_3_ with SiO_2_ nanoparticles (1 wt%), and NaNO_3_-KNO_3_ with SiO_2_ nanoparticles (1 wt%) and NaOH (0.03 wt%). Reprinted with permission from Ref. [26] Copyright 2020 Elsevier.

Evaluating the dispersion and stability of nanoparticles is crucial in understanding the properties of nanofluids. To assess thermal stability, some researchers have employed thermogravimetric analysis (TGA) and differential scanning calorimetry (DSC) curves [38,39]. TGA is a technique used in material science and analytical chemistry to analyze how a sample’s mass changes with temperature or time. It provides valuable insights into material heat stability and decomposition behavior. During TGA, a sample is continuously weighed while being heated or cooled in a controlled environment using a sensitive thermobalance that detects even the slightest mass changes. The sample and an empty reference pan are placed in the furnace, and, as the temperature rises at a constant rate, any mass loss due to evaporation, decomposition, oxidation, or other chemical processes is continuously monitored. The results are plotted as a thermogram, a graph depicting the mass change versus temperature or time. Figure 6 demonstrates that all samples in the study exhibited thermal stability up to 500 °C, with decomposition beginning only above this temperature. 

TGA boasts several advantages. It requires only a miniscule sample size (a few milligrams) and can analyze both solids and liquids with minimal preparation. However, TGA has some limitations. It is a destructive technique, meaning that the sample cannot be reused. Additionally, TGA cannot directly identify specific substances or detect chemical or physical changes that do not result in mass changes during heating. Despite these limitations, TGA offers valuable applications: (1) determining the thermal stability of materials; (2) quantifying the residual mass after heating; (3) identifying the moisture and volatile content of samples.

#### 3.1.3. T-History Method

**Figure 7:**
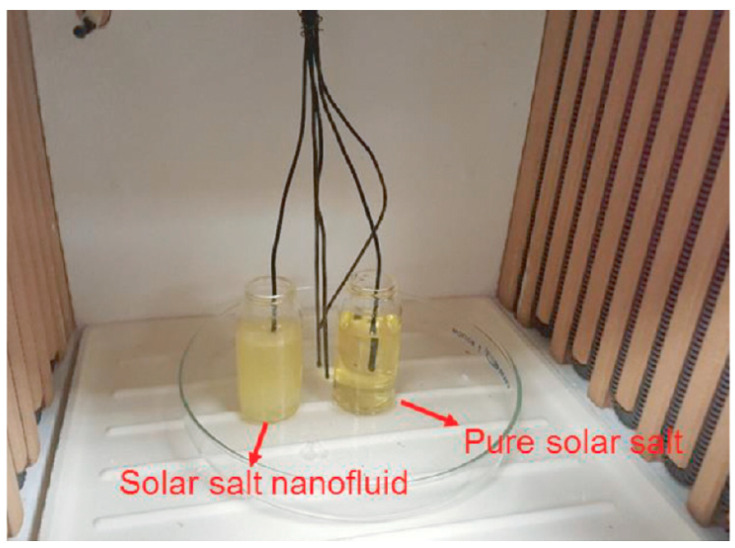
Experimental configuration of T-history test. Reprinted with permission from Ref. [33] Copyright 2021 Elsevier.

The T-history technique, previously introduced by [40], offers an alternative to DSC for the measurement of the thermal conductivity, heat fusion, and specific heat capacity (SHC) of phase transition materials. It allows for significantly larger sample sizes (>10 g) compared to DSC, potentially providing more representative and realistic results due to the larger sample volume. Ma et al. [33] employed the T-history technique to measure the SHC of their solar salt nanofluid. Figure 7 illustrates a basic T-history setup for heat measurements. It utilizes two vials: one containing a pure reference salt and the other containing the solar salt nanofluid, both with the same mass. Thermocouples measure the temperature of the molten salt in each vial, while additional thermocouples monitor the furnace temperature. The ratio of the SHC at any temperature is then calculated using Equation (1), where *m_ref_* and *m_nano_* are the masses of the reference sample and the nanofluid sample, respectively. The temperature versus time data are fitted with an appropriate model to obtain the rate of change in temperature with respect to time at any temperature, where *T_air_* and *T_s_* are the instantaneous temperatures of the furnace and sample, respectively.

(1) $$\frac{c_{p,nano}}{c_{p,ref}}=\frac{m_{ref}}{m_{nano}}\cdot \frac{{\left(\frac{\mathrm{d}T_{s}}{\mathrm{d}t}\right)}_{ref}}{{\left(\frac{\mathrm{d}T_{s}}{\mathrm{d}t}\right)}_{nano}}\cdot \frac{{\left(T_{air}-T_{s}\right)}_{nano}}{{\left(T_{s}-T_{air}\right)}_{ref}}$$

The T-history technique boasts several advantages over DSC. Firstly, it allows for the measurement of the heat fusion, thermal conductivity, and SHC simultaneously, providing a more comprehensive characterization of a material. Additionally, it can analyze larger samples (>10 g), potentially yielding results closer to the bulk material properties. Furthermore, T-history is often less complex and more cost-effective than DSC. However, T-history also has some limitations. It is not suitable for the study of kinetic phase transformations due to its slower cooling rates. The complex data analysis that it requires can be more intricate than DSC’s approach. Additionally, despite using larger samples, T-history may not always achieve the same level of accuracy as DSC. Finally, the need for larger samples can translate to higher costs in certain applications. Despite these limitations, T-history offers valuable functionalities: (1) measuring the specific heat capacity (SHC) of the sample; (2) determining the thermal conductivity of the sample; and (3) quantifying the heat fusion of the sample.

#### 3.1.4. Simultaneous Thermogravimetric Analysis (STA)

The simultaneous measurement of heat flow and mass change is also possible using STA. This technique eliminates the need for separate TGA and DSC analyses, reducing material consumption and minimizing potential discrepancies arising from using different instruments. STA’s ability to simultaneously measure both heat flow and mass change has been employed in several investigations [41,42] to characterize the MS nanofluid’s specific heat capacity and mass change. By using a single sample, STA conserves valuable material resources and avoids errors introduced by sample handling, preparation, and instrumentation differences. However, STA also has some limitations. Compared to DSC or TGA, it requires a more sophisticated instrument and may sometimes exhibit lower sensitivity to minor heat events. Despite these limitations, STA finds valuable applications in polymer characterization, material science and engineering, pharmaceutical analysis, food, and agriculture.

#### 3.1.5. Transient Hot Wire Method

**Figure 8:**
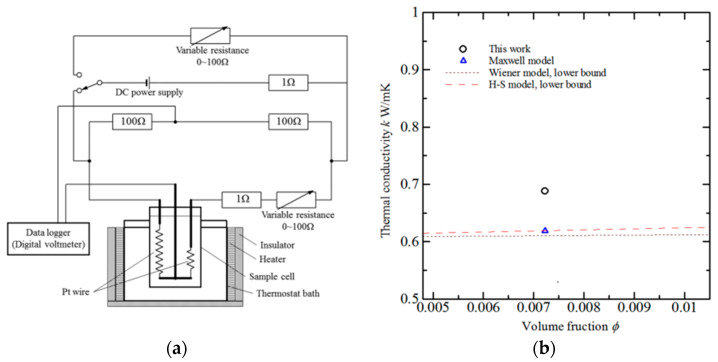
(**a**) Experimental setup of transient hot wire method and (**b**) thermal conductivity of the ternary molten salt obtained utilizing transient hot wire. Adapted from Ref. [43].

In the transient hot wire method, a thin wire with known thermal conductivity is submerged in a solution and heated with a direct current. This wire acts as both a sensor and a heat source. As heat dissipates from the hot wire to the surrounding molten salt, the wire’s temperature changes. By monitoring the temporal change in the wire’s temperature, scientists can determine the thermal conductivity of the molten salt. This technique was used in a previous study [43] to characterize the thermal conductivity of a ternary salt mixture (NaNO_2_-NaNO_3_-KNO_3_) doped with SiC, as shown in Figure 8b. Figure 8a depicts the experimental apparatus used in the study.

#### 3.1.6. Laser Flash Method

**Figure 9:**
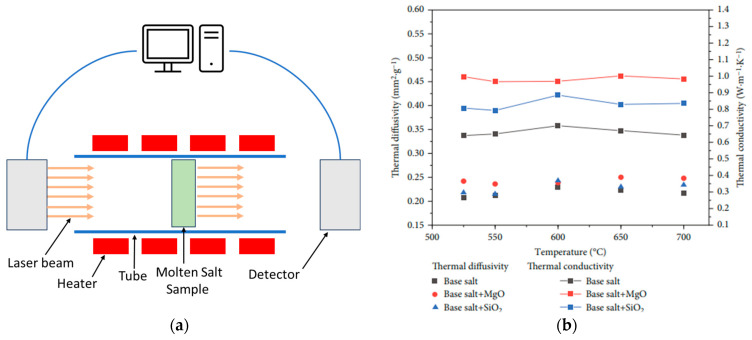
(**a**) Schematic of the laser flash technique and (**b**) the average thermal conductivity obtained. Adapted from Ref. [44].

Another technique for the measurement of thermal conductivity is laser flash. In this method, a powerful laser pulse heats the material from one side, while a detector on the other side monitors the temperature change over time. By analyzing the temperature rise and diffusion, researchers can calculate the material’s thermal conductivity. Figure 9a illustrates a schematic of the laser flash apparatus. Using the laser flash technique, Yaxuan et al. [44] investigated the impact of SiO_2_ and MgO additions on the thermal conductivity of the binary carbonate salt Li_2_CO_3_-K_2_CO_3_. Their findings revealed that the MgO-containing nanofluid exhibited the highest thermal conductivity enhancement, reaching a remarkable 55.7% increase compared to the pure salt mixture. Figure 9b showcases the results of their study, highlighting the significant improvement in thermal conductivity achieved through MgO addition.

#### 3.1.7. Customized Concentric Cylinder Method

**Figure 10:**
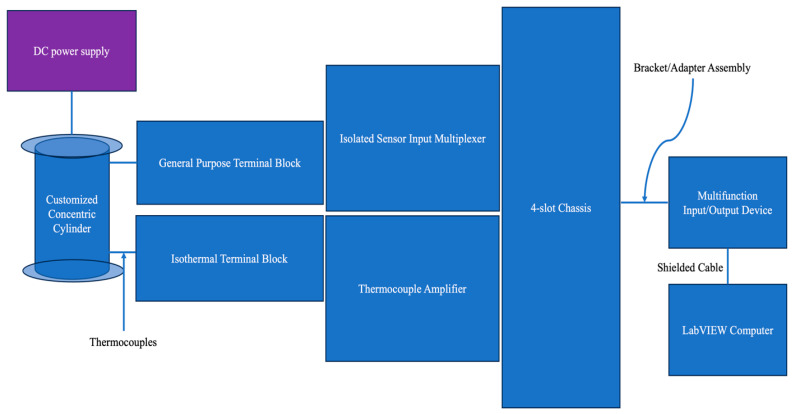
Schematic diagram of thermal conductivity measurement of MS nanofluids.

Understanding the heat transfer behavior of nanofluids is crucial, and thermal conductivity is a key parameter in this aspect. While the laser flash technique is popular for thermal conductivity measurements, its applicability is limited to the solid phase [45,46]. For molten salt eutectics, the transient hot wire method is more frequently utilized [7,47,48]. However, measuring the thermal conductivity of nanofluids like MS nanofluids using the hot wire technique poses challenges due to their nanoscale particles. When a nanoparticle, less than a nanometer in size, adheres to the hot wire, it acts as a miniature fin, significantly increasing the heat transfer rate. This poses challenges for accurate measurement. Additionally, the high temperatures associated with molten salts necessitate specialized equipment. To address these challenges, a previous study employed a custom-designed concentric cylinder apparatus to measure the thermal conductivity of both the molten salt eutectic and its nanofluid [49]. Figure 10 illustrates the setup and procedure for this method. The customized measurement system consists of the LabVIEW software, a data acquisition (DAQ) system, a DC power supply, K-type thermocouples, and a laboratory furnace. These include a shielded cable, a general-purpose terminal block, an isothermal terminal block, an isolated sensor input multiplexer, a thermocouple amplifier, a 4-slot chassis, a bracket/adapter assembly, and a multifunction input/output device.

### 3.2. Material Characterization

#### 3.2.1. Scanning Electron Microscopy (SEM)

**Figure 11:**
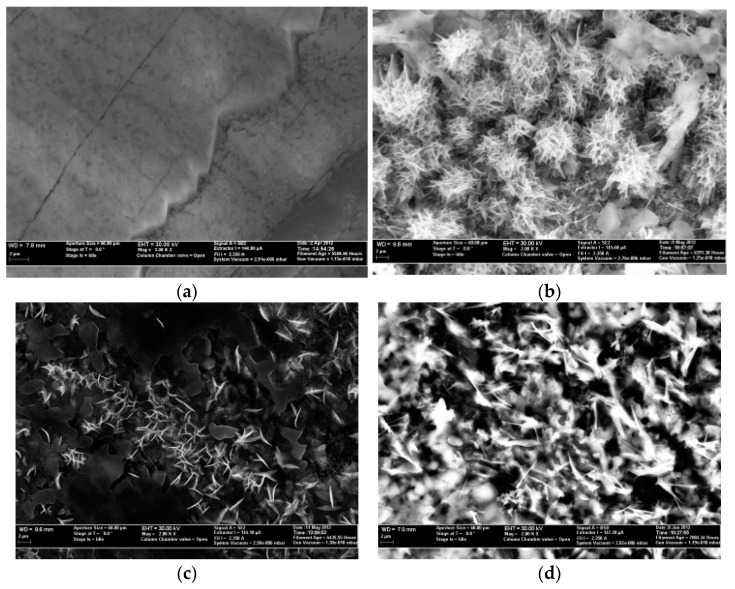
SEM images of (**a**) pure binary carbonate eutectic and binary carbonate nanofluid (**b**) with 5 nm nanomaterial structure, (**c**) with 10 nm nanomaterial structure, and (**d**) with 60 nm nanomaterial structure. Reprinted with permission from Ref. [37] Copyright 2013 Elsevier.

Empirical methodologies play a crucial role in analyzing nanofluids based on molten salts (MS). These methods provide valuable insights into the morphology, composition, and structure of these materials. One of the most widely used techniques for material characterization is scanning electron microscopy (SEM) [50]. SEM delivers high-resolution images of a specimen’s surface, enabling the detailed analysis of its morphology, particle size, and distribution. The technique works by focusing an electron beam on the specimen and detecting the emitted secondary electrons. These results offer valuable information about the nanofluid’s structural properties. For example, a previous study [37] used SEM to characterize both the pure eutectic salt (Figure 11a) and nanomaterials with different particle sizes (Figure 11b–d). Figure 11a shows an undifferentiated molten salt formation exhibiting typical characteristics. Conversely, Figure 11b–d reveal the presence of needle-like features throughout the nanomaterials, regardless of the particle size. These needle-like structures are expected to have a significantly higher specific surface area compared to the bulk eutectic. Figure 11d, obtained using an SEM backscattered electron (BSE) detector, highlights compositional differences. The varying contrast between the needle-like structures and the eutectic matrix indicates dissimilar salt compositions. This observation provides valuable information about the nanofluid’s structure and potential properties. These nanostructures have extremely large surface areas and are therefore likely to absorb moisture from the atmosphere. A moisture-free environment is necessary to avoid these structures being dissolved and returned to the eutectic when loading in SEM.

#### 3.2.2. Transmission Electron Microscopy (TEM)

**Figure 12:**
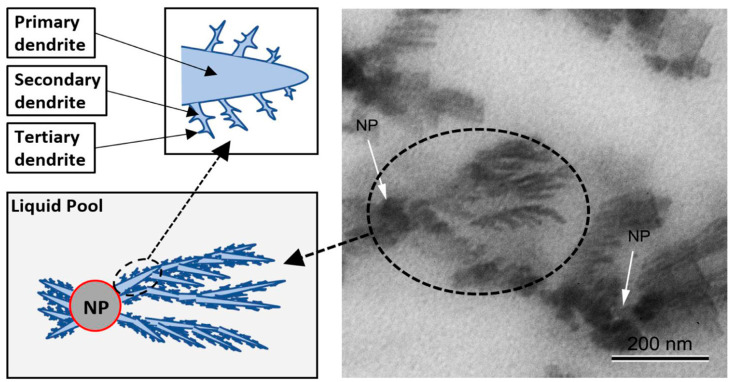
A schematic representation of dendrite formation alongside a transmission electron microscopy micrograph showcasing salt dendritic structures. Reprinted with permission from Ref. [51] Copyright 2020 Elsevier.

Transmission electron microscopy (TEM) offers an even higher resolution than SEM, allowing for the analysis of nanofluids at the atomic level. This technique works by directing an electron beam through a thin sample and detecting the transmitted electrons. The resulting image reveals the internal structure of the nanofluid, including the arrangement and size of individual nanoparticles. The superior resolution of TEM makes it invaluable in understanding the intricate details of the nanofluid’s morphology. For example, Rizvi and Shin [51] used TEM to visualize the formation of dendritic nanostructures in their MS nanofluid, as shown in Figure 12.

#### 3.2.3. Energy-Dispersive Spectroscopy (EDS)

**Figure 13:**
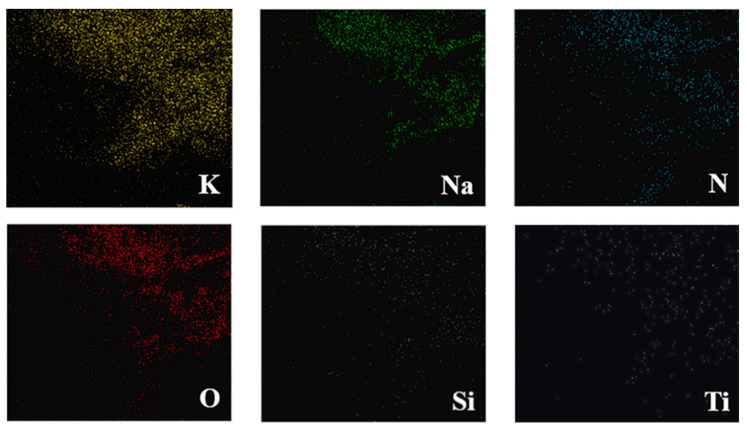
EDS mapping element of molten salt nanofluid containing 0.1 SiO_2-_0.9 TiO_2-_base fluid. Reprinted with permission from Ref. [42] Copyright 2021 Elsevier.

Scanning electron microscopy (SEM) and transmission electron microscopy (TEM) are often paired with energy-dispersive X-ray spectroscopy (EDS) to reveal the elemental composition and distribution within a material. This powerful technique works by bombarding the material with high-energy electrons, causing atoms to emit characteristic X-rays. An X-ray detector captures and analyzes these X-rays, providing detailed information about the elements present and their spatial distribution. EDS is a valuable tool for the characterization of molten salt nanofluids, as demonstrated in numerous studies. For example, Yu et al. [42] investigated the impact of adding two different nanoparticles (SiO_2_ and TiO_2_) to a quaternary nitrate salt mixture. To assess the homogeneity of the element distribution in the resulting nanofluid, they utilized EDS element mapping. As shown in Figure 13, the EDS map reveals the uniform distribution of the various elements within the nanofluid. This indicates the successful incorporation of the nanoparticles and suggests a well-mixed mixture. This information is crucial in understanding the nanofluid’s properties and potential applications.

#### 3.2.4. Fourier-Transform Infrared Spectroscopy (FTIR)

FTIR is used to characterize molten salts. By measuring the infrared radiation absorption depending on a molecule’s vibrational mode, FTIR makes it possible to determine the functional group inside a molecule. This approach enables bulk analysis (several micrometers) and does not need any previous sample preparation. Mondragon et al. [52] used FTIR to establish nitrate adsorption on the surfaces of nanoparticles. Although FTIR can be used to analyze the functional groups, it cannot provide information about the elemental composition.

#### 3.2.5. X-ray Diffraction (XRD) 

**Figure 14:**
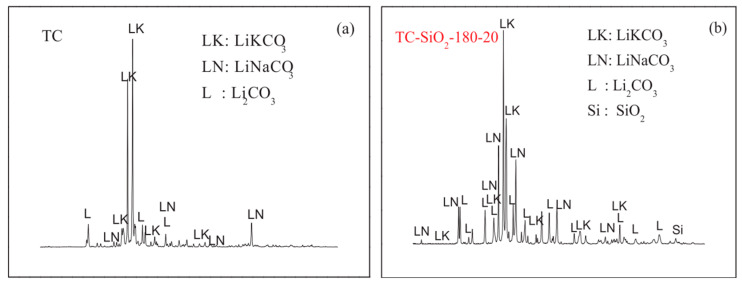
XRD diffraction patterns (**a**) before and (**b**) after adding nanoparticles (SiO_2_) with a size of 20 nm. Reprinted with permission from Ref. [41] Copyright 2017 Elsevier.

XRD is a powerful and non-destructive technique used to investigate the crystallographic structure of a material. It provides valuable information about the type of lattice, the unit cell dimensions, and the presence of different phases within the material. This is possible because X-rays, with their wavelength in the nanometer range (similar to the spacing between atoms), are scattered by the atoms in the material. By analyzing the resulting diffraction patterns, scientists can decipher the underlying crystal structure. XRD plays a crucial role in material research, and Sang and Liu [41] used it to study the interaction between nanoparticles and molten salts. Their experiment aimed to determine whether any chemical reaction occurred between the two. To achieve this, they analyzed the diffraction patterns of the molten salt before and after adding nanoparticles. Figure 14 showcases the results of their study. The plot comparison highlights that the original peaks in the diffraction pattern remained largely unchanged after introducing the nanoparticles. This indicates that no new phases or significant alterations to the crystal structure occurred. However, the emergence of new peaks attributable to SiO_2_ confirmed the successful incorporation of the nanoparticles into the molten salt. This observation, supported by the unchanged primary peaks in the diffraction pattern, provides strong evidence that no chemical interaction took place between the nanoparticles and the molten salt. This valuable information is crucial in understanding the compatibility and potential behavior of such composites in various applications. 

#### 3.2.6. Dynamic Light Scattering (DLS) and Zeta Potential Analysis

Dynamic light scattering (DLS) and zeta potential analysis are common methods of characterizing nanofluids, as demonstrated in several studies [53]. DLS analyzes the intensity fluctuations of light scattered by nanoparticles in a suspension, providing valuable insights into their hydrodynamic size and size distribution. Zeta potential analysis, on the other hand, measures the electrostatic charge on the nanoparticles’ surface. This information is crucial in assessing nanofluid stability, as the charge determines the strength of interparticle forces (attraction or repulsion) and therefore the tendency of the nanoparticles to clump together. While significant progress has been made in characterizing nanofluids, several challenges remain. Achieving and maintaining the homogeneous dispersion of nanoparticles in molten salt matrices, especially at high temperatures, is a major challenge. Additionally, accurately measuring the thermophysical properties at high temperatures can be difficult due to limitations in existing experimental setups and the potential reactivity of the nanofluids with the measurement equipment. Therefore, the careful planning and meticulous execution of experimental procedures are essential in ensuring the reliable and precise characterization of MS-based nanofluids [54].

## 4. Nanoparticle–Salt Interactions and Their Impact on Heat Transfer and Storage Capacity

### 4.1. Thermal Conductivity

Understanding the heat transfer behavior in nanofluids requires a comprehensive understanding of the interplay between the nanoparticles and the molten salt (MS) matrix. Nanoparticle introduction can significantly alter the MS’s thermal properties by modifying its local structure and dynamics. Furthermore, the heat transfer efficiency heavily relies on the nanoparticle dispersion and stability within the MS. Meanwhile, the interactions between the nanoparticles and salt molecules can improve the interfacial contact, leading to more efficient thermal energy transfer between the two. This enhanced contact facilitates effective heat conduction from the MS to the nanoparticles, ultimately boosting the nanofluid’s heat transfer performance. Mastering and manipulating these interactions is key to optimizing the thermal properties of MS-based nanofluids [50]. Table 2 demonstrates the thermal conductivity enhancement of MS nanofluids found in previous studies.

### 4.2. Specific Heat Capacity (SHC)

**Figure 15:**
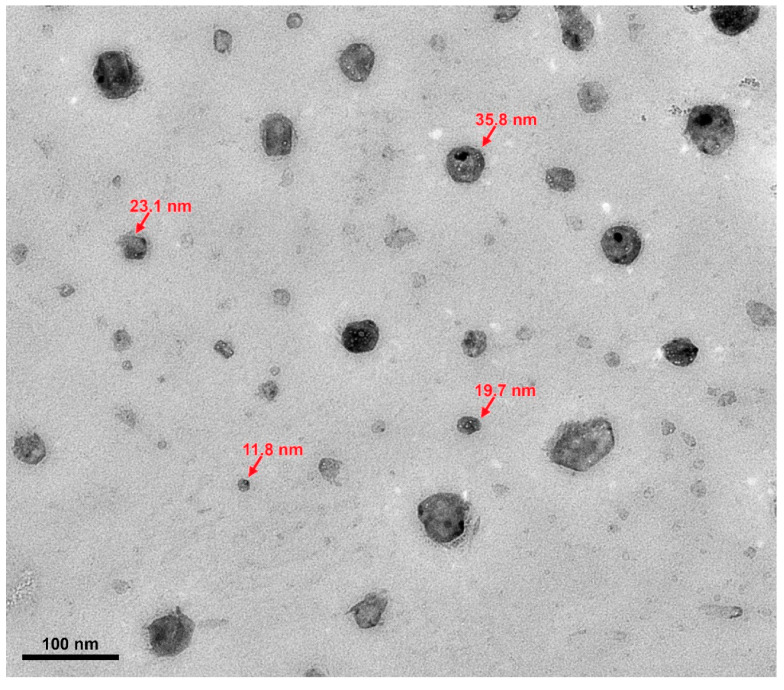
TEM micrograph of polydispersed Al_2_O_3_ NPs in MS nanofluids. Reprinted form Ref. [62].

Table 3 summarizes the highest recorded enhancements in the specific heat capacity (SHC) for various salt systems and synthesis techniques. These values, previously published in the literature, represent the peak SHC achieved for a specific nanoparticle–salt combination across varying concentrations and particle sizes. An analysis of Table 3 reveals that NaNO_3_-KNO_3_ (60:40 weight ratio) and Li_2_CO_3_-K_2_CO_3_ (62:38 molar ratio) were the most commonly used base salt systems. Among the most frequently employed nanoparticles, SiO_2_ and Al_2_O_3_ stood out. Interestingly, the solution-based method dominated nanofluid production. Nanoparticle diameters typically ranged from 10 to 20 nm. Furthermore, a 1% mass concentration of nanoparticles consistently yielded the highest SHC increases. Previous investigations have employed dynamic light scattering (DLS), scanning electron microscopy (SEM), and transmission electron microscopy (TEM) to characterize the size and shape of nanoparticles. As illustrated in Figure 15, the TEM micrograph of alumina nanoparticles generated using the in-situ approach reveals a size variation among the particles. Additionally, the image suggests a non-uniform shape distribution. These observations collectively support the conclusion that the nanoparticles exhibit polydispersity. Despite substantial research on SHC enhancement, understanding the underlying mechanisms remains a key challenge.

**Figure 16:**
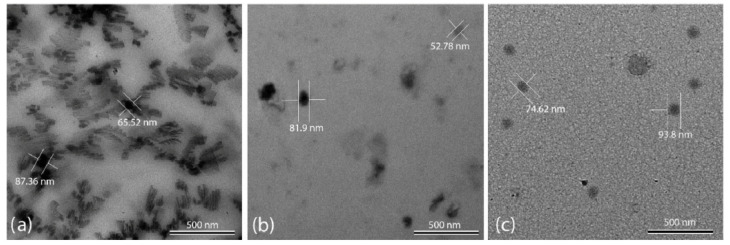
(**a**) The nanofluid forms dendritic nanostructures; (**b**) When the nanofluid is diluted with distilled water, the presence of nanoparticles and the absence of dendritic nanostructures verify that the nanostructures are composed of soluble salts; (**c**) The nanofluid is added with NaOH to prevent the formation of dendritic nanostructures. Reprinted with permission from Ref. [51] Copyright 2020 Elsevier.

While investigating the same nanofluids, different research teams sometimes report conflicting specific heat capacity (SHC) values. This discrepancy could be attributed to variations in their chosen synthesis techniques. However, even research groups employing the same synthesis protocol can sometimes encounter diverse SHC enhancements. Rizvi and Shin [51] shed light on this phenomenon by analyzing the nanofluid structure using TEM. They diluted the nanofluid with distilled water and examined the resulting dendritic nanostructures (Figure 16a) to determine their compositions. Figure 16b shows the same nanofluid after dilution, where the dendritic structures have vanished, revealing only individual nanoparticles. This observation, coupled with the known solubility of salts and insolubility of nanoparticles, confirms that the dendritic structures are primarily composed of salts. This finding holds significant implications for the reported discrepancies in SHC values (Table 3). Inadequate moisture management during synthesis can easily disrupt the formation of these beneficial dendritic structures, thereby limiting the potential SHC enhancement of the molten salt nanofluid. Figure 16c showcases a nanofluid treated with NaOH, demonstrating the absence of the dendritic structures observed in Figure 16a. Therefore, we can conclude that insufficient control over the pH and moisture levels during synthesis can hinder the formation of desirable nanostructures in MS nanofluids, ultimately reducing their SHC.

## 5. Heat Transfer Enhancement Mechanisms in MS Nanofluids

**Figure 17:**
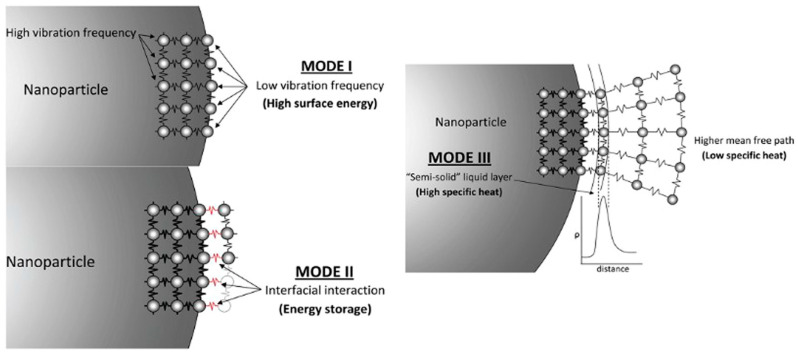
Models proposed by Shin and Banerjee for the enhancement of specific heat in MS nanofluids. Reprinted with permission from Ref. [30] Copyright 2011 Elsevier.

Despite significant research on specific heat capacity (SHC) enhancement in nanofluids, the underlying mechanisms remain largely unexplained. Shin and Banerjee [30] first proposed three possible explanations for the anomalous increase in SHC observed in molten salt (MS)-based nanofluids. (1) Enhanced SHC of nanoparticles: Smaller particles exhibit a higher theoretical SHC compared to their bulk counterparts due to their increased surface area and quantum confinement effects. (2) Interaction energy between solids and fluids: Interactions between nanoparticles and surrounding salt molecules can lead to an energy exchange that contributes to the overall SHC of the nanofluid. (3) Semisolid layer formation: The layering of liquid salt molecules around the nanoparticles might create a semisolid interface with unique thermal properties, impacting the overall SHC. These theoretical models aim to explain the unusual SHC behavior observed in MS nanofluids and provide a starting point for further investigation (Figure 17).

### 5.1. The Heightened SHC of Nano-Sized Particles

Mode I describes the enhanced specific heat capacity (SHC) observed in nanoparticles compared to their bulk counterparts (Figure 17). This theory proposes that as the particle size decreases, the confinement of surface atoms weakens. These unbound atoms gain more freedom to vibrate, leading to higher overall energy and, consequently, an increased SHC. This effect is more pronounced on the large surface areas of nanoparticles, contributing to a significant SHC boost. Experimental evidence supports this concept, with studies showing an increase of up to 25% in the SHC of alumina nanoparticles compared to their bulk material. This significant enhancement highlights the potential of this mechanism in contributing to the overall SHC improvement observed in molten salt nanofluids [30].

### 5.2. The Interaction Energy between Solids and Fluids

Mode II focuses on the “solid–fluid interaction energy” arising from the interactions between the nanoparticles and the molten salt (MS) (Figure 17). This interaction can benefit the nanofluid’s thermal storage capacity by introducing thermal resistance at the interface, known as Kapitza resistance. This Kapitza resistance stems from the vast surface area that nanoparticles present per unit mass. This extensive interface, negligible at larger scales, creates a unique thermal boundary between the solid nanoparticles and the liquid MS. This boundary can hinder heat transfer across the interface, effectively “trapping” thermal energy within the nanofluid, thereby boosting its capacity for thermal storage [81].

### 5.3. The Formation of a Semisolid Layer through the Layering of Liquid Molecules at the Surface

Mode III revolves around the intriguing phenomenon of “layering” observed at the interface between the nanoparticles and the molten salt (MS) (Figure 17). This theory proposes that the MS ions organize into a structured layer around the nanoparticles, forming a semisolid shell with unique thermal properties. This layer, estimated to be between 2 and 5 nanometers thick, exhibits significantly higher thermal characteristics compared to the bulk liquid. Remarkably, the existence of these semisolid layers has been confirmed through experimental observations using transmission electron microscopy (TEM), as reported in the scientific literature. These findings lend credence to the Mode III model and suggest its potential contribution to the enhanced SHC observed in MS nanofluids [82].

Several researchers have critically examined the models proposed by Shin and Banerjee, along with available experimental data [83]. Khanafer and colleagues [84] meticulously reviewed these models and emphasized the need for further empirical and theoretical investigations due to the observed discrepancies between research groups. Molecular dynamics simulations (MDS) have emerged as a valuable tool in understanding the behavior of MS-based nanofluids at the molecular level. Shin and Banerjee employed computational simulations to analyze the interfacial thermal resistance between carbon nanotubes and silica nanoparticles suspended in a carbonate eutectic mixture [30]. Their aim was to identify the optimal nanoparticle size to maximize the thermal conductivity. They also proposed a model to enhance the specific heat and used MDS to explore various nanofluids. The simulation results provided insights into the factors contributing to the increased specific heat in MS-based nanofluids, such as the formation of nanolayers and the higher concentrations of salt molecules surrounding the nanoparticles. These findings offer valuable information for further research and development efforts aimed at optimizing the thermal properties of these promising materials.

While promising, the impact of nanoparticles on the specific heat capacity (SHC) of molten salt (MS) nanofluids remains modest. Typically, these nanofluids contain only 1% nanoparticles by weight, resulting in a minimal overall effect on the SHC. Additionally, the potential contributions of interfacial thermal resistance and semisolid layering to SHC enhancement are still unclear. This ambiguity is further reinforced by the lack of significant SHC increases observed in conventional nanofluids. Doping nanoparticles into water, oil, or ethylene glycol often yields minimal SHC improvements. Prior research [85] identified agglomeration upon introducing nanoparticles into oils, yet no substantial SHC rise was observed. This suggests that agglomeration itself is unlikely to be the primary factor behind the SHC enhancement in MS nanofluids.

### 5.4. Ion-Exchange Mechanism

**Figure 18:**
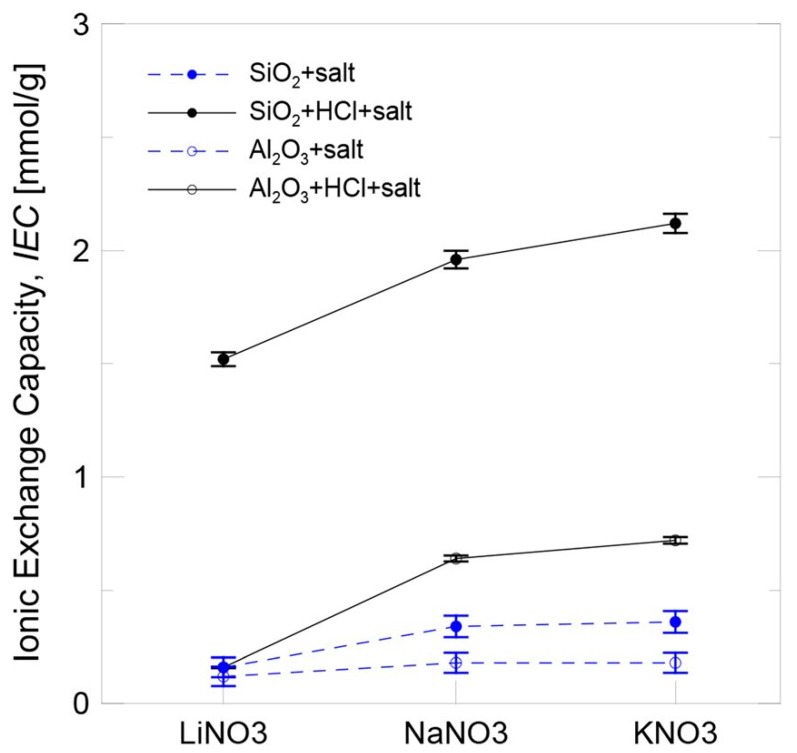
Ionic exchange capacity of NPs in salt solution. Adapted from Ref. [52].

In contrast, Mondragón and colleagues proposed that the enhancement of the SHC can be attributed to an ion-exchange mechanism occurring between nitrate ions and nanoparticles [52]. Four samples were produced for each case and subjected to two cycles of DSC analysis. Thus, the average of eight measurements was determined. The experimental error of the mean value was estimated using a 95% confidence interval. The researchers employed Fourier-transform infrared spectroscopy (FTIR) to corroborate their results. Figure 18 displays the outcomes acquired for the ion-exchange capacity (IEC) of the silica and alumina nanoparticles distributed in a salt solution using two methods: protonation by the addition of HCl and non-protonation. The electrical conductivity of the silica nanoparticles was consistently greater than that of the alumina nanoparticles in all instances. The ionic exchange phenomenon is influenced by the surfaces of nanoparticles. Among the nanoparticles, those with the smallest particle size and the greatest specific surface area (such as silica) had a greater IEC. Although the nanoparticles were not previously protonated, they exhibited a lower IEC. However, their IEC increased during protonation due to the addition of HCl. Acid activation resulted in an increased quantity of transferable H+ on the surface of the nanoparticles and also played a role in the ionic exchange process. It may be deduced that the chemical composition of the salt has an influence on the IEC, and that the larger the implicated cation (Li+, Na+, or K+), the higher the IEC.

**Figure 19:**
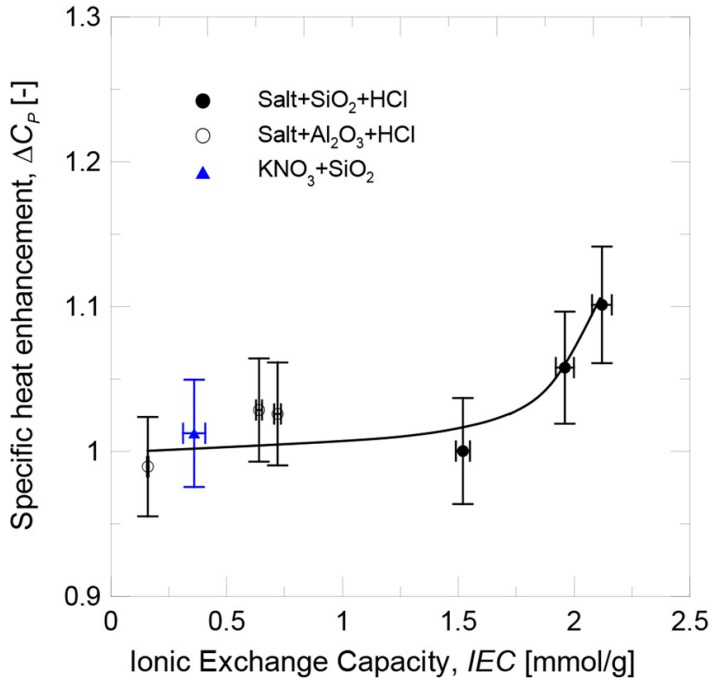
Progression of thermal capacity improvement by IEC. Adapted from Ref. [52].

With respect to the impact of the salt’s chemical composition, the specific heat enhancement for both nanoparticles became larger with the increasing cation size (Li+, Na+, and K+). The experimental circumstances (small particle size and larger cation size) that resulted in a greater increase in IEC were also characterized by a greater increase in specific heat enhancement. Thus, a correlation between the two was established (see Figure 19), and the effect of the ionic exchange process factors on the specific heat enhancement could be understood. The specific heat of nanofluids was not noticeably affected by the degree of functionalization of the nanoparticles for low IEC values. When the IEC was above a certain value, however, the highly functionalized nanoparticles began to contribute to an increase in specific heat enhancement.

### 5.5. Nanostructure Formation

Tiznobaik and Shin [83] proposed a new explanation for the observed SHC increase in MS nanofluids. They suggested that the enhanced specific surface energy of raised nanoparticle surfaces and the formation of needle-shaped structures contribute to this phenomenon. Additionally, another theory proposes that nano-dendritic structures formed around the nanoparticles can further enhance the SHC [86]. Rizvi and Shin [51] recently shed light on Tiznobaik’s findings regarding the growth of these dendritic structures, as shown in Figure 12. This image illustrates the development of primary, secondary, and even tertiary dendritic structures extending from the nanoparticles. Molecular dynamics simulations have further examined and validated these findings. Abir and Shin [31,87] used simulations to confirm the role of dendritic structures in SHC enhancement. However, these models could benefit from further refinement and development. Improved numerical modeling and simulations can shed light on the heat transport pathways within these high-SHC materials. This deeper understanding will ultimately aid in the design and optimization of real-world applications for MS nanofluids.

## 6. Conclusions and Future Suggestions

Concentrated solar power (CSP) offers a promising path towards a sustainable energy future. Its ability to generate clean, carbon-free electricity while being stable and easily dispatched makes it a compelling option. However, extensive research and development efforts are necessary to make CSP technology more efficient, cost-effective, and scalable. The high initial costs, particularly compared to other renewable technologies like photovoltaic (PV) systems, have posed a significant challenge for CSP adoption. Additionally, the deployment of CSP systems requires the careful consideration of several factors, including the availability of vast land areas, sufficient solar resources, and, in some designs, water for cooling. Molten salts (MSs) play a crucial role in certain CSP systems as a heat transfer and storage medium. They absorb and retain thermal energy, enabling continuous power generation even during off-sun hours. Recent advancements have spurred the exploration of MS nanofluids, which are suspensions of nanoparticles within MSs, as a potential means to enhance the heat transfer and thermal energy storage in CSP applications. Leveraging MS-based nanofluids for improved heat transfer and storage in CSP systems requires the careful consideration of several key factors.

Nanoparticle Selection: Nanoparticle selection is crucial in attaining enhanced heat transfer and storage properties. Thermal conductivity, stability at high temperatures, compatibility with molten ions, and the cost are factors to consider. Conductive nanoparticles, such as metallic or carbon-based nanoparticles, are frequently investigated for their high thermal conductivity, which can enhance the efficiency of heat transfer.

Nanoparticle Dispersion: Accomplishing the uniform dispersion of nanoparticles throughout the molten saline is essential for their efficient use. The agglomeration or sedimentation of nanoparticles can reduce their thermal conductivity. It is essential to develop techniques and additives that promote stable and uniform dispersion, such as surfactants and nanoparticle surface modifications.

Stability and Compatibility: Stability and compatibility are essential characteristics of nanofluids. The nanoparticles must remain dispersed and stable in the molten saline over extended periods of operation and at elevated temperatures. In addition, compatibility between the nanoparticles and the MS matrix is necessary to avoid chemical reactions or material degradation that could impact the system’s performance and lifespan.

Nanoparticle Selection: Choosing the appropriate nanoparticle is critical. Thermal conductivity, high-temperature stability, compatibility with molten salts, and cost are all crucial factors. Conductive nanoparticles, like metals or carbon-based materials, are often explored for their superior heat transfer capabilities.

Nanoparticle Dispersion: Evenly distributing nanoparticles throughout the molten salts is essential for optimal performance. Agglomeration or sedimentation can significantly reduce the efficiency. Developing techniques and additives, such as surfactants or surface modifications, to promote stable and uniform dispersion is crucial.

Stability and Compatibility: Long-term stability and compatibility are crucial for efficient operation. The nanoparticles must remain dispersed and stable in the molten salts over extended periods, even at high temperatures. Additionally, compatibility with the MS matrix is essential to avoid chemical reactions or material degradation that could affect the performance and lifespan.

System Integration and Safety: Integrating MS nanofluids into existing CSP systems demands the careful consideration of the design, material compatibility, and safety. Heat exchangers, piping, valves, and storage containers must be compatible with nanofluids and withstand high temperatures. Nanoparticle release and potential flow obstructions are hazards that must be mitigated through appropriate safety measures.

Economic Feasibility: Evaluating the cost-effectiveness of MS nanofluids is crucial for their practical implementation. Beyond potential performance benefits, the cost of the nanoparticles, the manufacturing processes, and scalability must be carefully assessed. An analysis of the cost-effectiveness can determine the viability of using nanofluids in real-world CSP applications.

Long-Term Durability: Long-term durability and stability are essential for reliable operation. Nanoparticle agglomeration, corrosion, erosion, and thermal degradation must be investigated over extended periods to ensure the continued performance and dependability of the CSP system.

Understanding the fundamental mechanisms of heat transfer enhancement in MS nanofluids is crucial. Several mechanisms, including increased thermal conductivity, enhanced convective heat transfer, and altered fluid flow characteristics, can contribute to enhanced heat transfer efficiency. Experimentation and computation can aid in the elucidation of these mechanisms and the optimization of nanofluid formulations.

To optimize the use of MS-based nanofluids for enhanced heat transfer and storage in CSP systems, research and development efforts continue to examine and resolve the aforementioned factors. It is possible to leverage the benefits of nanofluids while ensuring the safe and efficient operation of CSP facilities if these factors are thoroughly understood. Concerning nanoparticle dispersion, stability, material compatibility, safety, and cost-effectiveness, additional research is required. The realization of the great potential of MS nanofluids in CSP will rely heavily on ongoing research and advancements in nanofluid synthesis, characterization, and system integration.

Although there are still technical and economic obstacles to overcome, MS nanofluids’ future prospects in CSP are promising. As research advances and technologies mature, we can anticipate an increase in the efficacy, energy storage capacity, and deployment of nanofluid-based CSP systems, all of which will contribute to a more sustainable and renewable energy future.

## Figures and Tables

**Table 1:** Different combinations of base salt systems and nanoparticles used by different researchers.

Base Salt System	Nanoparticle (NP)	NP Size nm	NP%	Max. C_p_%	Ref.
NaNO_3_-KNO_3_ (60:40 wt.)	SiO_2_	15.2	1.0	15.00	[26]
KNO_3_	SiO_2_	20–25	4.0	15.70	[27]
NaNO_3_-KNO_3_ (60:40 wt.)	Al_2_O_3_	20	2.0	8.30	[28]
Li_2_CO_3_-K_2_CO_3_ (62:38 mole)	Graphite	-	1.0	14.3	[15]
NaNO_3_-KNO_3_ (60:40 wt.)	SiO_2_-Al_2_O_3_	2–200	1.0	22.50	[2]
NaNO_3_-KNO_3_-NaNO_2_ (7:53:40 mole)	Al_2_O_3_	40	0.063	19.90	[18]
K_2_CO_3_-Li_2_CO_3_-Na_2_CO_3_ (4:4:2 wt.)	SiO_2_	20	1.0	113.70	[29]
BaCl_2_-NaCl-CaCl_2_-LiCl (68.49:24.784:79.206:25.52)	SiO_2_	26	1.0	14.50	[30]

**Table 2:** Major thermal conductivity/diffusivity enhancements in MS nanofluids.

MS System	Nanoparticles	Best Concentration % wt.	Thermal Conductivity/Diffusivity Enhancement (%)	Ref.
NaNO_3_-KNO_3_	Silica	1.0	60.9	[55]
NaNO_3_-KNO_3_	Multi-Walled Carbon Nanotubes	0.3	293.0	[56]
Li_2_CO_3_-K_2_CO_3_	Single-Walled Carbon Nanotubes	1.5	57.0	[57]
MgCl_2_-KCl-NaCl	Alumina	0.7	48.0	[58]
Li_2_CO_3_-K_2_CO_3_-Na_2_CO_3_	Carbon Nanotubes	1.0	149.2	[59]
Li_2_CO_3_-K_2_CO_3_-Na_2_CO_3_	Magnesium Oxide	10.0	155.9	[60]
NaNO_3_-KNO_3_	Silica	1.0	50.0 (Diffusivity)	[55]
NaCl-CaCl_2_-MgCl_2_	Expanded Graphite	1.0	78.0	[61]

**Table 3:** Major SHC enhancements in MS nanofluids.

MS System	Nanoparticle (NP)	Synthesis Method	NP Size nm	NP%	Max. C_p_%	Ref.
NaNO_3_-KNO_3_ (60:40 wt.)	SiO_2_	LS [31]	12	1.0	25.03	[63]
Ca(NO_3_)_2_.4H_2_O-KNO_3_-NaNO_3_-LiNO_3_ (2:5:1:2 wt.)	SiO_2_	Mechanical dispersion	20	1.0	17.00	[64]
NaNO_3_-KNO_3_ (60:40 wt.)	SiO_2_	LS [31]	~15.2	1.0	15.00	[26]
NaNO_3_-KNO_3_ (60:40 wt.)	Al_2_O_3_	LS [31]	13	1.0	5.90	[2]
NaNO_3_-KNO_3_ (60:40 wt.)	SiO_2_-Al_2_O_3_	LS [31]	2–200	1.0	22.50	[2]
NaNO_3_-KNO_3_ (60:40 wt.)	SiO_2_-Al_2_O_3_	Direct method	2–200	1.0	18.60	[65]
Ca(NO_3_)_2_-KNO_3_-NaNO_3_-LiNO_3_ (2:6:1:2 wt.)	SiO_2_	Direct method	20	0.5	24.50	[66]
NaNO_3_-KNO_3_ (60:40 wt.)	SiO_2_	LS [31]	60	1.0	28.00	[67]
KNO_3_	SiO_2_	LS [31]	20–25	4.0	15.70	[27]
Li_2_CO_3_-K_2_CO_3_ (62:38 mol)	SiO_2_	LS [31]	2–20	1.5	124.00	[68]
NaCl-CaCl_2_ (52:48 mol)	Mg	Direct method	-	2.0	108.49	[69]
NaNO_3_-KNO_3_ (60:40 wt.)	Al_2_O_3_	LS [31]	20	2.0	8.30	[28]
Li_2_CO_3_-K_2_CO_3_ (62:38 mol)	CNT	LS [31]	10–30	1.0	29.30	[70]
NaNO_3_-KNO_3_ (60:40 wt.)	TiO_2_	In-situ method	16.35	3.0	7.50	[71]
NaNO_3_-KNO_3_ (60:40 wt.)	CuO	In-situ method	10-20	0.5	11.48	[72]
NaNO_3_-KNO_3_ (60:40 wt.)	SiO_2_	LS [31]	250+	3.0	9.70	[73]
NaNO_3_-KNO_3_ (60:40 wt.)	Al_2_O_3_	LS [31]	5~15	1.0	50.00	[33]
NaNO_3_-KNO_3_ (60:40 wt.)	Al_2_O_3_	In-situ method	-	1.0	30.00	[33]
NaNO_3_-KNO_3_ (60:40 wt.)	Al_2_O_3_	Freeze drying	10–20	2.0	12.33	[74]
NaNO_3_-KNO_3_ (60:40 wt.)	SiO_2_	Direct method	20	1.0	17.60	[75]
NaNO_3_-KNO_3_ (60:40 wt.)	TiO_2_	Mechanical dispersion	34	0.5	4.95	[76]
NaNO_3_-KNO_3_ (60:40 wt.)	Cuo	Mechanical dispersion	29	0.1	10.48	[76]
KNO_3_	SiO_2_	LS [31]	5–15	1.0	28.06	[24]
KNO_3_	SiO_2_	LS [31]	5–15	0.5	12.23	[24]
K_2_CO_3_-Li_2_CO_3_-Na_2_CO_3_ (4:4:2 wt.)	SiO_2_	LS [31]	20	1.0	113.70	[29]
K_2_CO_3_-Li_2_CO_3_-Na_2_CO_3_ (4:4:2 wt.)	SiO_2_	Mechanical mixing	20	1.0	38.50	[77]
Li_2_CO_3_-K2CO_3_ (61.0:39.0 mol)	CNT	Ball milling method	-	1.75	16.02	[78]
KNO_3_	Fe_2_O_3_	LS [31]	20–40	1.0	7.56	[79]
NaNO_3_-KNO_3_-NaNO_2_ (7:53:40 mol)	Al_2_O_3_	N_2_ gas stirred process	40	0.063	19.90	[80]
